# Managing mild autonomous cortisol secretion (MACS): evaluating the role of medical treatment

**DOI:** 10.3389/fendo.2026.1850728

**Published:** 2026-06-30

**Authors:** Ee Wen Loh, Miguel Debono

**Affiliations:** 1 Division of Endocrinology, Department of Medicine, Sarawak General Hospital, Kuching, Malaysia; 2 Department of Endocrinology, Sheffield Teaching Hospitals NHS Foundation Trust, Sheffield, United Kingdom

**Keywords:** adrenal, cortisol, medical treatment, metyrapone, mild autonomous cortisol secretion (MACS), mifepristone

## Introduction

Mild autonomous cortisol secretion (MACS) is defined by a post-dexamethasone serum cortisol >50nmol/L (>1.8 µg/dL) in the absence of clinical features of overt Cushing’s syndrome. It is characterized by cortisol excess, particularly during the nocturnal period (1, 2). With the rising incidence of adrenal incidentaloma, MACS is being identified more frequently as 20-50% of these patients demonstrate autonomous cortisol secretion (3). MACS is associated with an elevated risk of cortisol-related comorbidities including diabetes mellitus (DM), hypertension, dyslipidemia and low bone mineral density (BMD). Moreover, MACS has been linked with increased risk of cardiovascular events, mortality, frailty and fragility fractures (4–7). These associations underscore the importance of systematic evaluation and appropriate screening for potential complications in affected individuals as well as the importance of treating the condition.

## Benefits of surgery

Although treatment of the underlying etiology is fundamental, robust evidence defining the optimal therapeutic approach is still lacking. The latest European Society of Endocrinology guideline on management of adrenal incidentaloma suggests considering surgical intervention (adrenalectomy) in the presence of multiple excess cortisol-related comorbidities which are progressive and difficult to treat. This recommendation is based on findings of several studies demonstrating metabolic improvement following adrenalectomy. More recently, a multicenter randomized controlled trial (CHIRACIC study) involving 52 participants recruited between 2015–2022 reported a reduction in antihypertensive treatment with normal home blood pressure measurements (HBPM) in 46% of participants treated with adrenalectomy, compared to only 15% of those treated conservatively (8). Similarly, in the Co-work of Adrenal Research (COAR) study by Koh et al, patients in the adrenalectomy group exhibited significantly higher frequency of improved weight, glucose and blood pressure control compared with the control group (9). A meta-analysis further suggests benefit of adrenalectomy in patients with MACS (10). Nevertheless, due to limited evidence, clear criteria for surgical indication are lacking and decision regarding adrenalectomy should be made on an individual basis.

The decision between surgical referral and conservative management is not always straightforward. Adrenalectomy may not be appropriate for all patients, particularly those with contraindications to surgery, bilateral lesions or those who decline for surgical intervention. Moreover, a subset of patients may fail to achieve clinical improvement following surgery. For these individuals, current management has largely focused on optimizing control of associated comorbidities. However, recent findings from a retrospective, international cohort study suggest that this approach may be insufficient, highlighting the importance of directly targeting glucocorticoid excess to reduce morbidity and mortality (11). Accordingly, there has been growing interest in the potential role of medical treatment in this population to directly address excess cortisol.

## Study outcomes of medical treatment

Saini et al. reported that patients with MACS exhibit elevated evening serum cortisol level and a blunted circadian cortisol rhythm (12). Similar findings were observed by Debono et al. in a Phase 1/2a study of 6 patients with MACS and 12 control subjects, although a different diagnostic cut off was used (post-dexamethasone serum cortisol >80nmol/L or 60 to 80nmol/L with adrenocorticotropic hormone (ACTH) <2.2pmol/L) (1). Additionally, these patients showed elevated interleukin-6 (IL-6) levels, a pro-inflammatory cytokine involved in the pathogenesis of atherosclerosis; both abnormalities contribute to an increased cardiovascular risk (13, 14). Metyrapone, an 11*β-*hydroxylase inhibitor with a short half-life traditionally used in treatment of Cushing’s syndrome, was prescribed to patients with MACS at a dose of 500mg at 6pm and 250mg at 10pm. This regimen effectively lowered IL-6 and nocturnal cortisol level, thereby restoring a normal cortisol rhythm. Its short half-life allows daytime cortisol levels to remain largely unaffected while enabling more precise dose titration (1). A prospective proof-of-concept trial by Niziolek et al. using the same regime in 15 patients with MACS lowered hepatic lipid content and improved the metabolic risk parameters (15). Subsequent real-world studies by Berry et al. and Musolino et al. reported improved blood pressure control with Metyrapone therapy at doses of 250mg-750mg/day (administered in two divided doses at 6pm and 10pm) and 250-500mg/day (given in one or two divided doses at 1pm and 8pm), respectively. No cases of adrenal insufficiency were observed. The treatment was well tolerated, with most adverse effects being mild and transient (16, 17). However, larger randomized controlled trials are required to definitively ascertain efficacy and safety of this approach.

Another agent, Mifepristone has also been evaluated as a therapeutic option in patients with MACS in three separate studies. Debono et al. treated 6 patients with MACS with Mifepristone 200mg twice daily and this led to significant reduction in insulin resistance (18). Similarly, in a study involving 8 patients with MACS, treatment with Mifepristone 300mg daily for 3–6 months led to significant reduction in fasting plasma glucose levels and insulin resistance (19). More recently, in the interventional second phase of the CATALYST study, 136 individuals with endogenous hypercortisolism [post-dexamethasone serum cortisol >50nmol/L (>1.8 µg/dL) with or without adrenal abnormalities] and sub optimally controlled DM (mean HbA_1c_ 8.55%) were randomized to receive Mifepristone or placebo. Of these, 38 patients had adrenal abnormalities detected on computed tomography (CT) imaging. Treatment with Mifepristone resulted in a statistically significant reduction in HbA_1c_ of 1.47%, representing a 1.32% greater reduction than that observed in the placebo arm. Among patients with adrenal abnormalities, HbA1c decreased by 1.41%. Improvements in body weight, body mass index (BMI) and waist circumference were also observed across the study population (20). Additional therapeutic strategies are under investigation, including a Phase 2 study evaluating the impact of 1mg Osilodrostat in patients with MACS (21). Furthermore, an ongoing clinical trial is comparing Metyrapone and Osilodrostat for the treatment of MACS (22).

Despite these potential benefits, evidence supporting the impact of medical therapy on clinical outcome remains inconsistent. In an international retrospective study conducted by Nowak et al, 629 patients with hypercortisolism and bilateral adrenal tumors were evaluated, of whom 524 had MACS. Among patients with MACS, 8% (42 patients) received steroidogenesis inhibitors; although no significant improvement in comorbidities was observed, there was also no deterioration, except for osteoporosis. In contrast, patients who did not receive cortisol-targeted therapy experienced worsening of comorbidities (11). In addition, the potential adverse effects of these therapies must also be carefully considered. Inhibition of 11*β-*hydroxylase by Metyrapone leads to the accumulation of 11-deoxycortisol and 11-deoxycorticosterone, which may result in elevated blood pressure, thereby offsetting the therapeutic benefits of treatment. The accumulation of androgenic steroid precursors may also contribute to the development of hirsutism and acne, which can be particularly problematic in women. With Mifepristone, serum cortisol and ACTH would typically be elevated due to its glucocorticoid receptor antagonist activity. Consequently, biochemical monitoring of treatment response can be challenging, particularly in the identification of functional hypocortisolism. This complexity was highlighted in the aforementioned CATALYST study, in which a substantial proportion of patients discontinued Mifepristone during the study due to adverse events; among the 46% who discontinued treatment, many experienced symptoms potentially attributable to functional hypocortisolism (20). Additionally, its anti-progesterone effects increase the risk of endometrial hyperplasia.

## Conclusion

In conclusion, reduction of cortisol exposure for patients with ACTH-independent hypercortisolism is essential and medical therapy represents a potential treatment alternative for those who are not suitable candidates for adrenalectomy. Medical treatment may also play a role in identifying patients who are likely to benefit from surgery through preoperative monitoring of metabolic markers, as observed in individuals with Cushing’s syndrome (23). Patients who demonstrate clinical improvement with cortisol-lowering therapy could then be recommended for surgical intervention, unless contraindicated. However, despite emerging data, evidence supporting its efficacy remains limited. Further prospective studies are needed to establish the optimal diagnostic and therapeutic strategy roles of medical treatment in MACS, considering its potential side effects.
